# Functional evaluation of the apoptosome in renal cell carcinoma

**DOI:** 10.1038/sj.bjc.6601436

**Published:** 2003-11-25

**Authors:** M C Gerhard, N Zantl, G Weirich, S Schliep, B Seiffert, G Häcker

**Affiliations:** 1 Institute for Medical Microbiology, Immunology and Hygiene, Trogerstrasse 9, Munich D-81675, Germany; 2 Department of Urology, Ismaningerstrasse 22, Munich D-81675, Germany; 3 Institute of Pathology, Trogerstrasse 18, Technische Universität München, Munich D-81675, Germany

**Keywords:** renal cell carcinoma, apoptosome, caspases, IAP

## Abstract

Renal cell carcinoma (RCC) responds very poorly to chemo- or radiotherapy. Renal cell carcinoma cell lines have been described to be resistant to apoptosis-inducing stimuli and to lack caspase expression. Here, we provide a structural and functional assessment of the apoptosome, the central caspase-activating signalling complex and a candidate for apoptosis-inactivating mutations. Cells from RCC cell lines and clinical samples isolated from RCC patients were included. Apoptosome function was measured as quantitative activation of caspases in protein extracts. In all five cell lines and in 19 out of 20 primary clear cell RCC samples, the expression of apoptosome components and caspase activation appeared normal. Of the four nonclear cell RCC that could be included, both oncocytomas gave no response to cytochrome *c* (in one case, no Apaf-1 was detected), one chromophobe RCC lacked caspase-9 and failed to activate caspase-3 in response to cytochrome *c*, and one papillary RCC showed good caspase activation despite the lack of caspase-7. Experiments utilising a peptide derived from Smac/DIABLO gave no indication that inhibitor of apoptosis proteins might exert an inhibiting effect in primary clear cell RCC. Thus, the apoptosome signalling complex is intact in human (clear cell) RCC, and an apoptosis defect must be located at other, probably upstream, sites.

With its incidence varying significantly among countries, renal cell carcinoma (RCC) accounts for about 2% of all cancer cases worldwide (Motzer *et al*, 1996). Metastatic disease is often present at the time of diagnosis of RCC, and its poor response to radio- and chemotherapy determines its poor prognosis. Renal cell carcinoma is a heterogeneous disease and is classified by a combination of histopathology, histochemistry and molecular genetics. The majority of cases fall under the definition of clear cell carcinomas (also called conventional carcinomas, Kovacs *et al*, 1997, about 80%), followed by about 14% of papillary (=chromophilic) carcinoma (both probably originating from the proximal tubule) and about 3% of chromophobic carcinoma. The entity of renal oncocytic adenoma is considered a benign neoplasm (for review, of RCC, see Motzer *et al*, 1996).

Current understanding of tumorigenesis assumes that a multistep process of changes leads to the development of cancer. A number of ‘traits’ have been recognised as likely important in this process (Hanahan and Weinberg, 2000); these are among others a deregulation of cellular replication and an evasion of apoptosis (Hanahan and Weinberg, 2000; Green and Evan, 2002). Cell death by apoptosis occurs when the intracellular apoptotic pathway is activated, a conserved signal transduction system (Vaux and Strasser, 1996). Signals inducing apoptosis can be very diverse and encompass the direct stimulation of certain plasma membrane molecules (especially the so-called death receptors), the lack of stimulation through receptors (growth factor withdrawal, serum starvation) or less well-defined insults such as treatment with chemicals or irradiation, commonly referred to as ‘cellular stress’ (for review see Vaux and Strasser, 1996; Green and Evan, 2002). A great number of details are known about the signal transduction of apoptosis (see below).

The data are unequivocal on the finding that experimental inhibition of apoptosis can contribute to tumour development, as first demonstrated 15 years ago. Although the inhibition of apoptosis by itself is normally not sufficient to cause tumours, experimental prevention of apoptosis in mice could massively contribute to tumour development when proliferation was concomitantly enhanced (Vaux *et al*, 1988; Strasser *et al*, 1990). This indicates that apoptosis serves as a defence mechanism to avoid tumour development. The ability to evade apoptosis, which may result from genetic alterations, will thus enhance the cell's propensity to malignancy. Evasion of apoptosis is conceivable on a number of levels, from changes in the perception of signals to mutations in the apoptotic pathway proper. Many components and principles of the apoptosis system are known. The core of the pathway consists of proteases of the caspase family. Caspase-3 (to a lesser extent caspases-6 and -7) serves to cleave cellular protein substrates and thereby to bring about the apoptotic phenotype. Caspase-3 can either be activated directly by caspase-8 (upon caspase-8 activation through death receptors) or by a signalling complex referred to as ‘apoptosome’, consisting of cytochrome *c*, Apaf-1, caspase-9 (Li *et al*, 1997) and, at least sometimes, the X-linked inhibitor of apoptosis protein (XIAP; Bratton *et al*, 2001). In the apoptosome, cytochrome *c* serves to allow oligomerisation of Apaf-1 and thereby of caspase-9. Caspase-9 is activated in this process (Li *et al*, 1997; Zou *et al*, 1999). X-linked inhibitor of apoptosis protein has the potential to inhibit active caspase-3 and can thus halt or slow down the process at this step (Bratton *et al*, 2001). Cytochrome *c* is normally sequestered in the mitochondria of the cell. Its release into the cytosol during apoptosis is thus a crucial step of the apoptotic signal transduction. Although it is still not entirely clear how this release is regulated, the role of members of the Bcl-2 protein family in this process is well established (Adams and Cory, 2001; Kuwana *et al*, 2002). Three functionally different classes within this family of these proteins are known, apoptosis-inhibiting proteins (for instance, Bcl-2 itself), proteins that effect the release of cytochrome *c* (Bax and Bak) and a group of structurally relatively distant family members, the so-called BH3-only proteins (Huang and Strasser, 2001). A number of theories about the molecular function of these proteins in apoptosis have been put forward. The arguably most promising (although likely incomplete) model at present is that apoptotic stimuli activate one or more BH3-only proteins that then activate Bax/Bak, which in turn effect the release of cytochrome *c* (by a mechanism that is not understood). Bcl-2-like proteins in this model protect against apoptosis by sequestering active BH3-only proteins. For a recent discussion of this subject see Newmeyer and Ferguson-Miller (2003).

It has been demonstrated that a set of cell lines derived from human RCC almost completely lacked the expression of caspase-3 and expressed further caspases only to low levels (Kolenko *et al*, 1999). This suggested that loss of the expression of central caspases was a common feature in RCC, an alteration that might be expected to contribute to RCC development.

The present study was undertaken to follow-up on this finding in both cell lines and, more importantly, primary tumour material. We focused here on the analysis of the ‘apoptosome’ in these cells. Since inactivating mutations will not necessarily be apparent when only the presence of a protein is analysed, we also assayed the functional competence of this signalling complex. Cells and cell extracts were prepared from tumour material and normal kidney tissue that had been freshly isolated from patients undergoing nephrectomy for RCC. The expression of the components of the apoptosome as well as the response to cytochrome *c* were analysed, and the inhibitory potential of endogenous inhibitor of apoptosis proteins (IAP) was assessed.

## MATERIALS AND METHODS

### Cell lines and isolation of primary cells

The following human cell lines were from the tumour bank at the Deutsches Krebsforschungszentrum Heidelberg, Germany: KTCTL-1 M, KTCTL-26A, KTCTL-30 (all clear cell carcinoma) and KTCTL-84 (sarcomatoid carcinoma). CAKI-1 (clear cell carcinoma) was from ATCC (Rockville, MD, USA). Cells were cultured in complete Click's RPMI supplemented with 5% FCS, 5 mM glutamine and antibiotics in plastic culture dishes. At about 80% confluency, cultures were split into fresh plates. Primary cells were obtained from freshly explanted kidneys from patients undergoing nephrectomy for tumour treatment. In all, 24 patients (10 female, 14 male, age range 47–81 (median age 69) years) were included. The study was approved by the Klinikum rechts der Isar (Technical University Munich) ethics committee and patients gave their written consent to the use of their tumour tissues for research purposes. Diagnoses were: two oncocytomas, one papillary carcinoma (grading G1), one chromophobe carcinoma (G2) and 20 clear cell carcinomas (12 G2, eight G3). Both macroscopically normal and malignant tissue samples (about, 1 g each) were excised within 1 h of nephrectomy. Cells were isolated by collagenase digestion (3 mg ml^−1^ collagenase in complete Click's RPMI at a volume/weight ratio of 3) for 2–3 h at 37°C on a rocker. The resulting cell suspension was passed through a metal mesh (200 *μ*m width) and washed three times in complete medium. Cells were layered in a medium onto a discontinuous Biocoll gradient (Biochrome, Berlin; two phases: 75 or 100% in PBS) and centrifuged for 30 min at 400 **g**. The fraction at the medium–75% interface was used for experiments (in some cases, when not enough cells could be recovered from this step, cells from the 75 to 100% interface were also included). An aliquot from these cells was spun on glass slides, stained (May–Grünwald and Giemsa) and evaluated microscopically. Composition of the preparation was assessed by microscopy. Only tumour samples that contained at least 70% of discernible intact tumour cells (20 out of a total of 36 tumours processed) were included in the study (the remainder being stroma cell, debris and cells whose morphology had been distorted in the process so as to make a cytologic diagnosis impossible).

### Extract preparation

Cells were washed in PBS and resuspended in extraction buffer (S-100 buffer: 20 mM HEPES-KOH (pH 7.5), 10 mM KCl, 1.5 mM MgCl_2_, 1 mM Na–EDTA, 1 mM Na–EGTA, supplemented with 2 mM DTT and a protease inhibitor cocktail (Roche, Mannheim, Germany)) at 10^8^ cells ml^−1^. Cells were lysed by one cycle of freeze–thawing (liquid nitrogen, thawing at room temperature) and lysates were cleared by centrifugation at 4°C at 10 000 **g** for 10 min. Protein concentration was measured as absorption at 280 nm (it was normally around 50 mg ml^−1^). Extracts were stored at −80°C.

### Caspase activation by cytochrome c

Reactions were set up in 40 *μ*l and contained 20 mg ml^−1^ extract protein in S-100 buffer supplemented with 2 mM DTT and dATP (1 mM). To some aliquots cytochrome *c* was added to 1 or 50 *μ*g ml^−1^, and reactions were incubated at 37°C for 1 h. After this incubation, the induced effector caspase activity was measured as the activity that cleaved the fluorigenic caspase peptide substrate Ac-Asp-Glu-Val-Asp-7-amido-4-methyl-coumarin (DEVD-AMC). For this assay, triplicate reactions were run in a 96-well flat-bottom plate and contained 10 *μ*l of the above mixture and 90 *μ*l of reaction buffer (50 mM NaCl, 2 mM MgCl_2_, 40 mM *β*-glycerophosphate, 5 mM EGTA, 0.1% CHAPS, 100 *μ*g ml^−1^ BSA, 10 mM HEPES (pH 7.0), 10 *μ*M (final concentration) DEVD-AMC). Reactions were incubated at 37°C and measurements for free AMC were taken over 40 min at 5 or 10 min intervals. Linear regression analysis was performed of the maximum increase of AMC fluorescence (during the first 5–20 min after the addition of DEVD-AMC). The slope of the resulting straight line was considered effector caspase activity. As a standard, a sample of extract prepared from the epitheloid cell line HeLa was included in every experiment (always from the same batch of HeLa extract). Results for kidney cell samples are given as percentage of this standard.

### Immunoblotting

An aliquot (10 *μ*l, corresponding to 200 *μ*g of total protein) of the cytochrome *c*-induced reaction was boiled in Laemmli buffer, run on 12% SDS–PAGE, and proteins were transferred onto a nitrocellulose membrane. The following primary antibodies were used for protein detection: anticaspase-3 (Pharmingen No. 556425), anticaspase-7 (Pharmingen No. 66871A), anticaspase-9 (Pharmingen No. 68086E), anti-APAF-1 (Pharmingen No. 68076E) and anti-*β*-actin (Sigma). Secondary antibodies were peroxidase-labelled anti-mouse or anti-rabbit goat antibodies (Jackson labs). Blots were developed using a chemiluminescence reaction (Perkin-Elmer). The expression of caspases/Apaf-1 was judged visually from blots that contained the same amount of protein of total HeLa cell extract alongside RCC samples. Semiquantitative assessment of expression was as follows: (0) no detectable expression; (1) levels less than; (2) levels equal to; and (3) levels higher than in HeLa cells. Caspase processing by cytochrome *c* was judged from the ratio of procaspase and mature caspase on Western blots. Semiquantitative values were allocated as follows: (0) no processing detectable; (1) processed caspase visible but no visible loss of procaspase; (2) decrease in the signal of the procaspase band visible; and (3) procaspase completely disappeared.

### Measurement of the inhibitory activity of IAP

A synthetic peptide corresponding to the seven N-terminal amino acids from the mitochondrial IAP inhibitor Smac/DIABLO (AVPIAQK; Chai *et al*, 2000; peptides were custom synthesised at Thermo Hybaid, Ulm, Germany) was added to the extracts. Incubation with cytochrome *c* and detection of caspase activity were performed as above. The assay was validated by titrating bacterially expressed GST-XIAP (the vector was a kind gift from Dr David Vaux, Melbourne) against the DIABLO peptide and a control peptide of the same sequence, but the reverse orientation (KQAIPVA).

## RESULTS

### Caspase activation by cytochrome *c* in cell lines from RCC

In the initial step, we analysed the integrity of the apoptosome in five RCC cell lines that had originally been established from separate human tumours (four clear cell RCC, one sarcomatoid carcinoma). As it has been reported for other RCC cell lines, these cells showed very little apoptosis when treated with classical apoptosis-inducing agents such as staurosporine, when compared for instance to HeLa cells (data not shown). It was our purpose not only to test for the presence of its components, but also for the proper function of the apoptosome. We therefore prepared protein extracts from the cells and investigated the capacity of exogenously added cytochrome *c* to activate caspases in these extracts *in vitro*. In this experimental system, apoptosome formation and the activation of caspase-9, -3, -6 and -7 occur very likely in the same fashion as they would upon the release of cytochrome *c* from the mitochondria in intact cells (Liu *et al*, 1996; Zou *et al*, 1999). The induction and detection of caspase activity in this approach therefore allows the assessment of the integrity of the apoptotic apparatus downstream of cytochrome *c* release. As a standard of a normal apoptosome extracts from the well-investigated human cell lines, HeLa (epitheloid) or Jurkat (T cell) were prepared and assayed in parallel.

**Figure 1:**
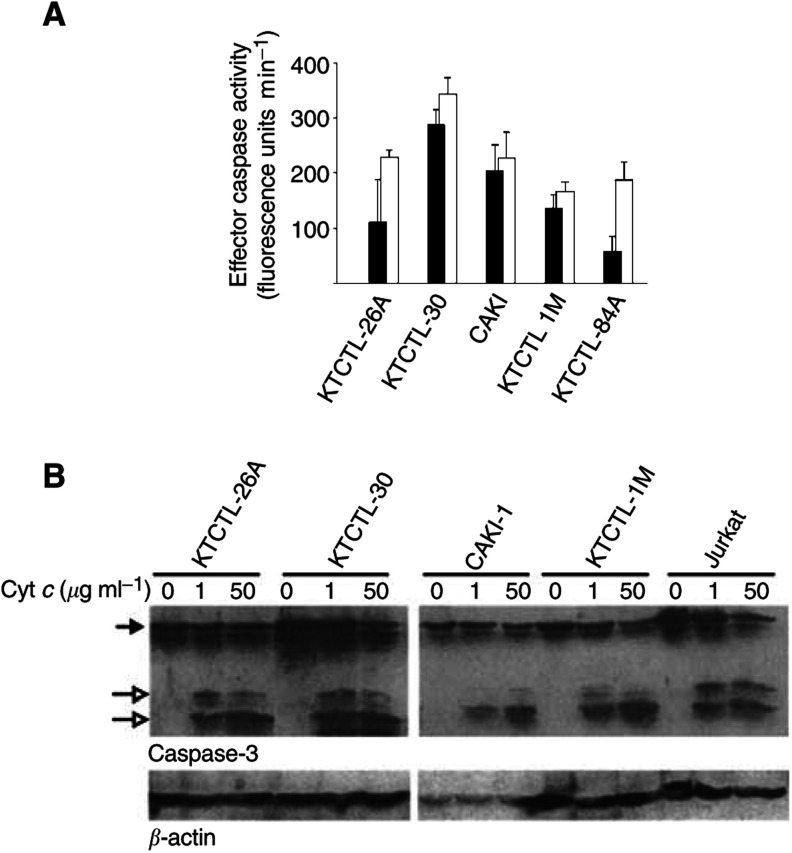
Composition and function of the apoptosome in five RCC cell lines. (**A**) Extracts from five RCC cell lines were incubated for 1 h at 37°C in the presence of cytochrome *c* (one (filled bars) or 50 (open bars) *μ*g ml)^−1^. Then, DEVD-cleaving activity was measured as release of AMC from the effector caspase-substrate DEVD-AMC (see Materials and Methods for details of the method). Columns give means and s.d. of the results from three separate experiments, each with separately prepared extracts. (**B**) The presence and activation of caspase-3 in RCC cell line extracts, extracts from the T-cell line Jurkat for comparison. Extracts were incubated with cytochrome *c* as above, and caspase-3 and *β*-actin were detected by immunoblotting (200 *μ*g of total protein per lane was loaded). Filled arrow, procaspase-3, open arrows, processed caspase-3. Results for caspase-3, -7 and -9 are summarised in Table 1.

The expression of caspase-3, -7 and -9 as well as Apaf-1 was normal by this criterion in all five cell lines as investigated by Western blotting (data not shown). Upon incubation of the extracts in the presence of cytochrome *c*, DEVD-cleaving (effector caspase) activity was detectable in all cases, and processing of caspase-3, -7 and -9 was observed in extracts from all five cell lines (Figure 1B

and Table 1

**Table 1:** Summary of caspase processing induced by cytochrome *c* in extracts from RCC cell lines

	**KTCTL-26A**		**KTCTL-30**		**CAKI-1**		**KTCTL-1M**		**KTCTL-84A**	
Cytochrome *c* (μg ml^−1^)	**1**	**50**	**1**	**50**	**1**	**50**	**1**	**50**	**1**	**50**
Caspase-3	0/1/1	2/1/2	1/1/2	2/2/2	1/1/1	2/1/2	1/1/1	2/2/2	1/1/1	1/2/1
Caspase-9	1/2	3/3	3/3	3/3	2/3	3/3	3	3	2/2	3/3
Caspase-7	1/3/3	3/3/3	3/3/3	3/3/3	3/3/3	3/3/3	3/3/3	3/3/3	2/3/1	3/3/3

Extracts from five RCC cell lines were incubated in the absence of cytochrome *c* or in the presence of 1 or 50 *μ*g ml^−1^ cytochrome *c* for 1 h at 37°C. Reactions were then tested by immunoblotting for processing of caspase-3, -7 and -9. Semiquantitative values were allocated as follows: (0) no processing detectable; (1) processed caspase visible but no visible loss of procaspase; (2), decrease in the signal of the procaspase band visible; and (3), procaspase completely disappeared. Experiments were performed three times for caspase-3 and -7, one or two times for caspase-9. Each value represents one separate experiment.

). The sensitivity towards cytochrome *c* appeared to be somewhat different between the cell lines, but they all gave a good reaction (i.e. caspase activation) at high concentrations of cytochrome *c* (Figure 1A). Apoptosome formation and function appears thus to be normal in the RCC cell lines investigated.

### Cytochrome *c*-induced caspase activation in primary renal cells

Taken together, the above results suggested that, in the cell lines investigated, the formation and activation of the apoptosome were normal. We then turned to the investigation of these parameters in human primary RCC. The protocol we devised was the following: when patients underwent nephrectomy, samples of both macroscopically normal and tumourous tissues were collected within 1 h of explantation. From these samples, cells were isolated and the quality of the cell preparation was controlled by staining and microscopical assessment. Extracts were prepared immediately following the isolation of the cells. The histopathologic composition of samples included was: clear cell carcinoma, 20 cases; papillary carcinoma, one case; chromophobe carcinoma, one case; and oncocytoma (nonmalignant), two cases. At the same time, cells were isolated by the same protocol from nontumourous tissue (five cases; referred to as normal cells).

In one sample from normal tissue no detectable DEVD-cleaving activity was induced by cytochrome *c* addition. Renal cell carcinoma material form the same patient (#65) was also defective in caspase activation (see below, Table 3). We were unable to determine the reason for this. Unfortunately, not enough material was available from this sample to repeat the experiment or to test the expression of Apaf-1. One possibility that would explain the lack of cytochrome *c*-driven caspase activation is a reduced expression of Apaf-1. It is also conceivable (although there is no evidence for this) that a technical problem during the preparation of the sample is responsible.

In extracts from the remaining four samples both activity and caspase processing could be detected to varying degrees with only one peculiarity: in one tissue sample, no caspase-7 was detected by immunoblotting while strong DEVD-cleaving activity was induced (Table 2

**Table 2:** Induction of DEVD-cleaving activity and caspase processing induced by cytochrome *c* in normal kidney cells

		**Caspase-3**		**Caspase-7**		**Caspase-9**		**Apaf-1**
**Patient**	DEVD-cleaving activity* (% standard)	**Expr.**	**Proc.**	**Expr.**	**Proc.**	**Expr.**	**Proc.**	**Expr.**
**#65**	0.8	++	0	+++	0	+	0	NT
**#66**	38.8	++	2	+	3	+	1	NT
**#70**	65.0	++	1	+	2	++	2	++
**#71**	197.9	++	2	−	0	++	2	++
**#78**	32.0	+	2	+	2	+	2	NT

Extracts from five samples of isolated normal kidney cells were incubated with cytochrome *c* (50 *μ*g ml^−1^) as above for 1 h at 37°C. DEVD-cleaving activity was measured and is shown as percent of HeLa cell extract control. Processing of caspases and the presence of Apaf-1 in extracts was assessed by immunoblotting. Semiquantitative assessment of processing was as follows: (0) no detectable expression; (1) levels less than; (2) levels equal to; and (3) levels higher than in HeLa cells (see also Materials and Methods). NT=not tested.

* DEVD-cleaving activity, effect of caspase activity (see Methods).

).

Somewhat surprisingly, there was no detectable defect in the expression and function of the apoptosome in extracts from clear cell RCC. Of the 20 isolates of which we had obtained satisfactory material, all but one showed a good activation of caspases by cytochrome *c* (unfortunately, we were unable to obtain information of this one as to expression of Apaf-1 and expression/activation of caspase-9). Caspase-3 was expressed to a significant level in all cases, and in all cases where we were able to test it the expression of the apoptosome components appeared normal (the results are summarised in Table 3

**Table 3:** Summary of caspase processing and effector caspase activity induced by cytochrome *c* in extracts from primary RCC samples

			**Caspase-3**		**Caspase-7**		**Caspase-9**		**Apaf-1**
**Patient #**	**Tumour**	DEVD-cleaving activity* (% standard)	**Expr.**	**Proc.**	**Expr.**	**Proc.**	**Expr.**	**Proc.**	**Expr.**
**36**	cc	85.2	++	1	+	3	+	2	+
**38**	cc	101.1	++	2	+	3	+	2	+
40	cc	123	+	2	+	3	+	NT	+
43	cc	85.3	+	2	+	3	+	NT	+
**45**	cc	116.4	++	2	+	3	+	3	+
47	cc	31.4	+	1	NT	NT	NT	NT	+
**48**	cc	34.5	+	2	NT	NT	NT	NT	+
49	cc	102.7	++	3	NT	NT	NT	NT	+
51	cc	87.1	++	2	NT	NT	NT	NT	+
**54**	cc	35.9	++	2	+	3	+	3	+
**55**	cc	30.7	++	2	+	2	+	3	+
57	cc	41.1	++	2	+	2	+	3	NT
**62**	cc	25.3	++	1	+	1	+	3	NT
64	cc	47.6	+	2	NT	NT	NT		
65	cc	2.0	++	0	NT	NT	NT		
**71**	cc	59.5	+	2	+	2	+	3	+
75	cc	69.3	+	2	NT	NT	+		
76	cc	90.7	+++	2	+	3	+	NT	+
77	cc	53.1	++	2	+	3	++	NT	+
78	cc	56.4	++	2	+	3	++	NT	+
Nonclear cell									
32	cp	3.55	+	0	+	0	−	0	+
39	oc	0.7	+	0	+	0	+	0	−
41	oc	1.2	+	0	+	0	++	0	+
72	prcc	67.7	++	2	−	1	+	0	NT

Extracts from 20 samples of clear cell RCC and four nonclear cell renal tumours were evaluated for the expression and function of apoptosome components (cc, clear cell; cp, chromophobe; oc, oncocytoma; prcc, papillary/chromophilic). Numbers of samples where tumour cell purity was above 90% appear in bold. Extracts were incubated in the absence of cytochrome *c* or in the presence 50 *μ*g ml^−1^ cytochrome *c* for 1 h at 37°C. Reactions were then tested by immunoblotting for processing of caspase-3, -7 and -9. Relative effector caspase activity is given as percent of HeLa control (assessment of the expression and processing was carried out as in Tables 1 and 2). NT=not tested (usually because of limitations of material).

**Figure 2:**
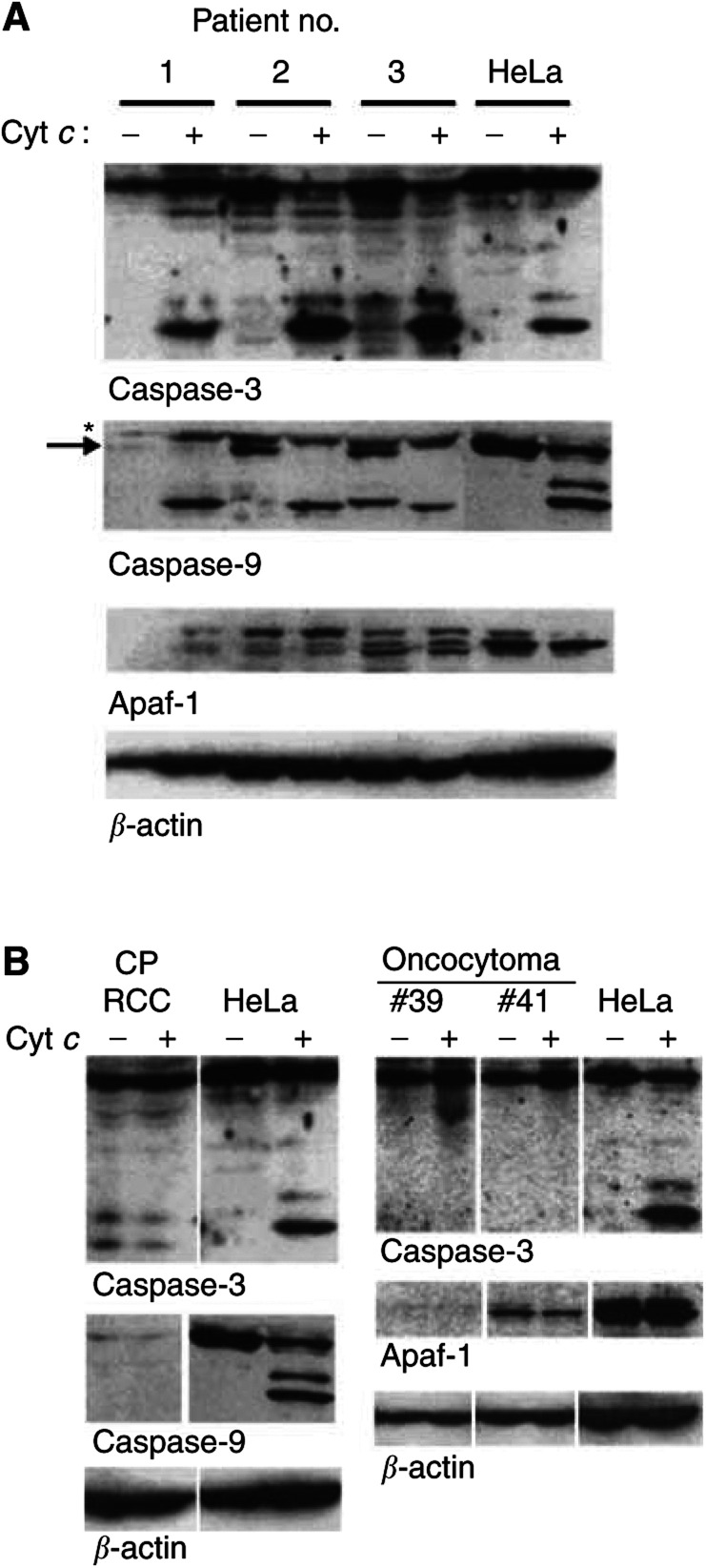
Examples of expression and processing of components of the apoptosome in clinical samples from RCC. Cells were isolated and extracts were prepared from fresh explants of clear cell RCC (**A**) or from one chromophobe RCC and two oncocytomas (**B**). Extracts from HeLa cells were prepared as above. Extracts (800 *μ*g protein in 40 *μ*l) were incubated for 1 h at 37°C in the presence or absence of 50 *μ*g ml^−1^ cytochrome *c*. A measure of 200 *μ*g per lane were run on SDS–PAGE and proteins were detected by Western blotting. Purity of the chromophobe RCC was about 80% tumour cells, suggesting that a contamination of 20% nontumour cells does not distort the results. (**A**) Asterisk denotes a nonspecific band, arrow procaspase-9. The several bands recognised by the anti-Apaf-1 antibody were seen in several experiments and may constitute Apaf-1 variants or, at least in part, products of nonspecific degradation. The smaller size band in the lane patient #3, no cytochrome *c* in the caspase-9 blot is of unknown origin and was not see in any other blot.

* DEVD-cleaving activity, effect of caspase activity (see Methods).

, and an example of caspase processing is given in Figure 2A

). It can thus be stated that, based on the analysis of 20 primary samples, the clear cell RCC contain a functionally competent apoptosome.

Although the sample size was too small to arrive at a definite conclusion, striking abnormalities were found in other entities of renal tumours (Table3 and Figure 2). Two (benign) oncocytomas, one chromophobe RCC and one papillary RCC could be included. In neither oncocytoma cytochrome *c* activated caspases. In one case, this defect correlated with a lack of Apaf-1 expression (Figure 2). The chromophobe RCC lacked the expression of caspase-9, which probably accounts for the complete absence of cytochrome *c*-induced caspase activation (Figure 2). In the case of papillary RCC (despite a nondetectable expression of caspase-7), the induction of DEVD-cleaving activity by cytochrome *c* was normal (Table 3). We conclude that at least in some cases of nonclear cell RCC the expression of apoptosome components is altered.

### Role of IAP on cytochrome *c*-dependent caspase activity

Members of the family of IAPs have the capacity to inhibit caspases. More precisely, they seem to target active caspase-9 or -3 and block its activity (Verhagen *et al*, 2001). A high level of expression of various IAP can therefore inhibit apoptosis at the level of the apoptosome and thus contribute to a resistance to apoptotic stimuli. The activity of the known antiapoptotic IAP can be overcome by the cellular protein Smac/DIABLO (reviewed in Silke and Vaux, 2001). DIABLO is normally sequestered in the mitochondria but is, like cytochrome *c*, released into the cytosol during apoptosis where it then acts to relieve the block in the apoptotic pathway imposed by IAP.

**Figure 3:**
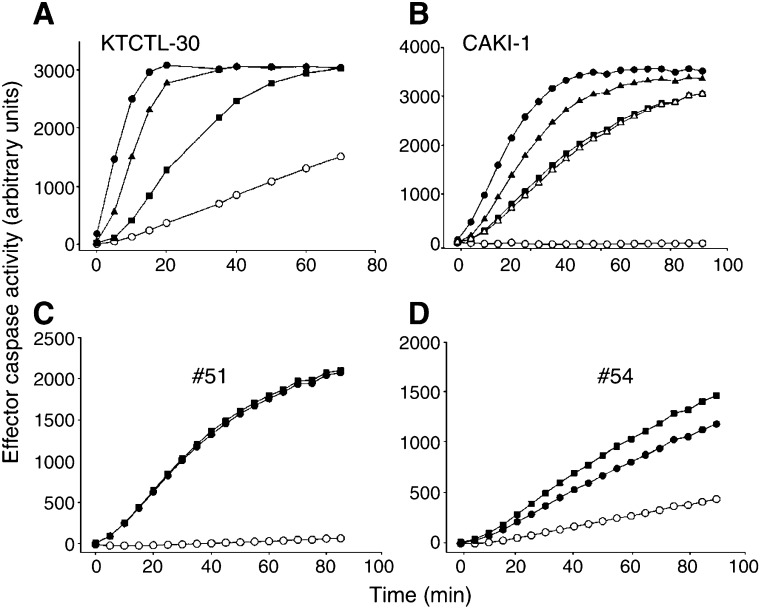
Effect of IAP on the cytochrome *c*-dependent generation of effector caspase activity. Extracts were prepared from the cell lines KTCTL-30 or CAKI-1 (top) or primary clear cell RCC (two examples are shown at the bottom). (**A** and **B**) Extracts were incubated without cytochrome *c* (open circles) or with cytochrome *c* (closed circles). To some samples recombinant XIAP was added together with cytochrome *c* and was found to inhibit the generation of caspase activity (squares). To evaluate the efficacy of DIABLO peptide, one sample was incubated with cytochrome *c*, XIAP and DIABLO peptide (300 *μ*M); DIABLO at least partially relieved the inhibition by XIAP (triangles). In (**B**), a control peptide of the same composition but the reverse orientation was added instead of the DIABLO peptide (300 *μ*M; together with cytochrome *c* and XIAP, open triangles). Effector caspase activity was measured as free AMC every 5 min, individual measurements are shown. (**C** and **D**) Examples of the effect of DIABLO peptide in extracts from primary RCC. Samples from patients (#51 and #54, see Table 3) were incubated without cytochrome *c* (open circles), with cytochrome *c* (50 *μ*g ml^−1^, closed circles) or with cytochrome *c* plus DIABLO peptide (300 *μ*M, filled squares). Effector caspase activity was measured as free AMC as above.

We utilised this principle to test for the possibility that a high level of IAP contributes to apoptosis resistance in RCC. The DIABLO protein can be functionally replaced by a synthetic peptide corresponding to its seven N-terminal amino acids; addition of this peptide to cell extracts has been shown to be able to revert the inhibition of IAP (Chai *et al*, 2000). Since various IAP could principally contribute to apoptosome inhibition and mere IAP expression levels may not be as informative, we compared the caspase activity induced by cytochrome *c* in the absence and the presence of DIABLO peptide. The difference between the two activities should correspond to the inhibition imposed by endogenous IAP. Initial experiments with extracts from RCC cell lines confirmed that recombinant purified XIAP reduced the DEVD-cleaving activity induced by cytochrome *c* in cell extracts. When DIABLO peptide was added, the activity was restored indicating that the peptide indeed was able to abolish the inhibitory activity of the added XIAP (Figure 3A

). A peptide of the same composition but the reverse orientation had no activity (Figure 3B). To test the degree of inhibition by endogenous IAP, we then added DIABLO peptide together with cytochrome *c* to extracts from RCC. In all, 12 samples of clear cell RCC (from the above collective) were tested in this way. In one sample, the addition of DIABLO peptide together with cytochrome *c* had a somewhat enhancing effect on the resulting DEVD-cleaving activity (Figure 3D), in the other 11 samples no effect could be detected (one example is shown in Figure 3C). It is thus unlikely that an IAP-imposed block on apoptosome function is a functionally important feature of clear cell RCC.

## DISCUSSION

The data presented in this study provide evidence that the apoptosome (i.e. the caspase-activating complex that is formed upon release of cytchrome *c*) is structurally and functionally intact in cells from RCC cell lines and primary RCC, at least when investigated by the addition of exogenous cytochrome *c*. Although, with the method used, a population of totally pure primary tumour cells could not be obtained, eight out of 20 samples contained over 90% of tumour cells, and this appears as a purity that is likely high enough to obtain results relevant to the tumour cell population. Alterations in this central structure are therefore probably not involved in this case of tumorigenesis or in the high level of resistance in these cells to cytotoxic stimuli.

It has been clear for over a decade that the inhibition of apoptosis can contribute to the development for cancer, a finding first demonstrated in mice overexpressing both *c*-myc (driving the cell cycle) and Bcl-2 (which inhibits apoptosis) in the lymphatic system (Strasser *et al*, 1990). Therefore, if not inhibited, apoptosis must occur during transformation and act to prevent tumour development. What the precise triggers of apoptosis during tumorigenesis are is not known. Under certain circumstances the overexpression of oncogenes, as it would occur in arising tumours, can trigger apoptosis (Fearnhead *et al*, 1997; Evan and Littlewood, 1998), and it may be such dysregulations of cellular functions that have to be countered by apoptosis defects. As already mentioned, mutations in the apoptotic apparatus may also be expected to contribute to increased resistance to radio- and chemotherapy.

Accordingly, apoptosis deficiencies can potentially arise at any step of the apoptotic pathway that is relevant to the initiation and implementation of apoptosis either during tumorigenesis or chemotherapy. The central compound of the apoptosis pathway, the apoptosome, is a plausible candidate for a target where mutations might have a tumour-promoting effect, although this may apply not to all cell types. A recent study shows that a loss of Apaf-1 or caspase-9 does not prevent apoptosis in cells of the haematopoietic system and does not rescue the clonogenic potential upon treatment with cytotoxic agents (Marsden *et al*, 2002); this means that mutations in the apoptosome are unlikely to contribute to malignant transformation in this cell type. On the other hand, sympathetic neurons can survive apoptosis induction and release of cytochrome *c* as long as caspases are inhibited experimentally; when the apoptotic stimulus is removed, the cells seem able to recover their full physiological capacities (Martinou *et al*, 1999). That inactivating mutations in the apoptosome can indeed contribute to tumour development has most impressively been demonstrated in a study that linked the loss of Apaf-1 to the development of malignant melanoma (Soengas *et al*, 2001). Whether such alterations would promote tumorigenesis in renal cells is unknown.

A number of chromosomal alterations are known to occur with high frequencies in RCC, such as deletions of chromosome 3p in clear cell RCC (Presti *et al*, 1991), trisomy 7, and trisomy 17 in chromophilic RCC and others (Kovacs *et al*, 1991). In approximately 40% of sporadic clear cell RCC, the inactivation of the von Hippel–Lindau (*VHL*) tumour suppressor gene located on chromosome 3p25 has been observed and seems to play a major role for the development of this tumour type (Foster *et al*, 1994, Gnarra *et al*, 1994, Brauch *et al*, 2000, Mandriota *et al*, 2002). The tumor suppressor function of *VHL* was further substantiated by the finding that reintroduction in a cell line blocked its ability to form tumours (Tamm *et al*, 2003). The loss of VHL -protein again results in the dysregulation of hypoxia-induced factor 1 (Maxwell *et al*, 1999; Mack *et al*, 2003), and this dysregulation may affect the sensitivity of the cells to apoptotic stimuli at least in some cell types (for a discussion of this subject see Piret *et al*, 2003). Comparative studies of VHL-positive and -negative RCC lines have found that VHL afforded a protection against hypoxia, possibly via upregulation of Bcl-2 (Devarajan *et al*, 2001).

The irregularities we describe here in oncocytoma and chromophobe RCC are interesting, but can have only anecdotal value at this stage. Oncoytoma is considered a benign tumour. This does not exclude the possibility that, for instance, a loss of Apaf-1 could contribute to tumour development. Likewise, the loss of caspase-9 expression in chromophobe carcinoma may contribute to the rise of this type of tumour, but more data are obviously needed to confirm this initial finding.

We believe that we can say with some certainty that in clear cell RCC the apoptosome is functionally intact. These data fail to confirm earlier findings derived from a number of cell lines that RCC cells have a severely reduced expression of caspases-3 (Kolenko *et al*, 1999). The resistance to apoptotic stimuli in RCC cells has been documented in cell lines (Wu *et al*, 2000) and, as already discussed, is likely to extend to primary tumours. Now we have excluded the cytochrome *c*-dependent apparatus, we will focus on the events at and upstream of mitochondria. A recent comprehensive study of gene expression in RCC provides much information about mRNA expression levels in primary tumours (Boer *et al*, 2001). Although a number of ‘apoptosis-related’ genes in that study have been found to have a changed expression in RCC, this appears to relate largely to genes that have functions in the broader context of apoptosis induction (i.e. proteins whose impact on the intracellular equilibrium helps to determine the live-or-die decision, such as k-ras (Arber *et al*, 1997), klotho (Nagai *et al*, 2003) or thioredoxin (Baker *et al*, 1997)) rather than genes whose products make up the apoptosis pathway itself (for instance, Bcl-2-family members). It certainly remains a distinct possibility that alterations other than a change in the expression of the intact gene account for apoptosis defects. Small structural mutations in critical apoptosis regulators could have profound effects on apoptosis resistance. It is our belief that further functional tests in the upstream apoptosis system are, at present, the most promising approach to advance our understanding of the tumour's behaviour with reference to apoptosis. Such tests should especially include the assessment of the resistance of mitochondria to activated BH3-only proteins (for instance, by using recombinant proteins in permeabilised cells); this would reveal a ‘mitochondria’ block in the apoptotic pathway. The assessment of the activation of BH3-only proteins upon treatment with apoptosis-inducing stimuli (such as the induction of Noxa and Puma proteins by ionising irradiation or the activation of Bim by UV irradiation, Huang and Strasser, 2000) might also reveal specific defects in the signal transduction of apoptosis.

## References

[bib1] Adams JM, Cory S (2001) Life-or-death decisions by the Bcl-2 protein family. Trends Biochem Sci 26: 61–66 11165519 10.1016/s0968-0004(00)01740-0

[bib2] Arber N, Han EK, Sgambato A, Piazza GA, Delohery TM, Begemann M, Weghorst CM, Kim NH, Pamukcu R, Ahnen DJ, Reed JC, Weinstein IB, Holt PR (1997) A K-ras oncogene increases resistance to sulindac-induced apoptosis in rat enterocytes. Gastroenterology 113: 1892–1900 9394728 10.1016/s0016-5085(97)70008-8

[bib3] Baker A, Payne CM, Briehl MM, Powis G (1997) Thioredoxin, a gene found overexpressed in human cancer, inhibits apoptosis *in vitro* and *in vivo*. Cancer Res 57: 5162–5167 9371519

[bib4] Boer JM, Huber WK, Sultmann H, Wilmer F, von Heydebreck A, Haas S, Korn B, Gunawan B, Vente A, Fuzesi L, Vingron M, Poustka A (2001) Identification and classification of differentially expressed genes in renal cell carcinoma by expression profiling on a global human 31,500-element cDNA array. Genome Res 11: 1861–1870 11691851 10.1101/gr.184501 PMC311168

[bib5] Bratton SB, Walker G, Srinivasula SM, Sun XM, Butterworth M, Alnemri ES, Cohen GM (2001) Recruitment, activation and retention of caspases-9 and -3 by Apaf-1 apoptosome and associated XIAP complexes. EMBO J 20: 998–1009 11230124 10.1093/emboj/20.5.998 PMC145489

[bib6] Brauch H, Weirich G, Brieger J, Glavac D, Rodl H, Eichinger M, Feurer M, Weidt E, Puranakanitstha C, Neuhaus C, Pomer S, Brenner W, Schirmacher P, Storkel S, Rotter M, Masera A, Gugeler N, Decker HJ (2000) VHL alterations in human clear cell renal cell carcinoma: association with advanced tumor stage and a novel hot spot mutation. Cancer Res 60: 1942–1948 10766184

[bib7] Chai J, Du C, Wu JW, Kyin S, Wang X, Shi Y (2000) Structural and biochemical basis of apoptotic activation by Smac/DIABLO. Nature 406: 855–862 10972280 10.1038/35022514

[bib8] Devarajan P, De Leon M, Talasazan F, Schoenfeld AR, Davidowitz EJ, Burk RD (2001) The von Hippel–Lindau gene product inhibits renal cell apoptosis via Bcl-2-dependent pathways. J Biol Chem 276: 40599–40605 11514546 10.1074/jbc.M103424200

[bib9] Evan G, Littlewood T (1998) A matter of life and cell death. Science 281: 1317–1322 9721090 10.1126/science.281.5381.1317

[bib10] Fearnhead HO, McCurrach ME, O'Neill J, Zhang K, Lowe SW, Lazebnik YA (1997) Oncogene-dependent apoptosis in extracts from drug-resistant cells. Genes Dev 11: 1266–1276 9171371 10.1101/gad.11.10.1266

[bib11] Foster K, Prowse A, van den BA, Fleming S, Hulsbeek MM, Crossey PA, Richards FM, Cairns P, Affara NA, Ferguson-Smith MA, Buys CM, Maher ER (1994) Somatic mutations of the von Hippel–Lindau disease tumour suppressor gene in non-familial clear cell renal carcinoma. Hum Mol Genet 3: 2169–2173 7881415 10.1093/hmg/3.12.2169

[bib12] Gnarra JR, Tory K, Weng Y, Schmidt L, Wei MH, Li H, Latif F, Liu S, Chen F, Duh FM (1994) Mutations of the VHL tumour suppressor gene in renal carcinoma. Nat Genet 7: 85–90 7915601 10.1038/ng0594-85

[bib13] Green DR, Evan GI (2002) A matter of life and death. Cancer Cell 1: 19–30 12086884 10.1016/s1535-6108(02)00024-7

[bib14] Hanahan D, Weinberg RA (2000) The hallmarks of cancer. Cell 100: 57–70 10647931 10.1016/s0092-8674(00)81683-9

[bib15] Huang DC, Strasser A (2000) BH3-Only proteins-essential initiators of apoptotic cell death. Cell 103: 839–842 11136969 10.1016/s0092-8674(00)00187-2

[bib16] Kolenko V, Uzzo RG, Bukowski R, Bander NH, Novick AC, Hsi ED, Finke JH (1999) Dead or dying: necrosis versus apoptosis in caspase-deficient human renal cell carcinoma. Cancer Res 59: 2838–2842 10383143

[bib17] Kovacs G, Fuzesi L, Emanual A, Kung HF (1991) Cytogenetics of papillary renal cell tumors. Genes Chromosomes Cancer 3: 249–255 1958590 10.1002/gcc.2870030403

[bib18] Kovacs G, Akhtar M, Beckwith BJ, Bugert P, Cooper CS, Delahunt B, Eble JN, Fleming S, Ljungberg B, Medeiros LJ, Moch H, Reuter VE, Ritz E, Roos G, Schmidt D, Srigley JR, Storkel S, van den BE, Zbar B (1997) The Heidelberg classification of renal cell tumours. J Pathol 183: 131–133 9390023 10.1002/(SICI)1096-9896(199710)183:2<131::AID-PATH931>3.0.CO;2-G

[bib19] Kuwana T, Mackey MR, Perkins G, Ellisman MH, Latterich M, Schneiter R, Green DR, Newmeyer DD (2002) Bid, bax, and lipids cooperate to form supramolecular openings in the outer mitochondrial membrane. Cell 111: 331–342 12419244 10.1016/s0092-8674(02)01036-x

[bib20] Li P, Nijhawan D, Budihardjo I, Srinivasula SM, Ahmad M, Alnemri ES, Wang X (1997) Cytochrome c and dATP-dependent formation of Apaf-1/caspase-9 complex initiates an apoptotic protease cascade. Cell 91: 479–489 9390557 10.1016/s0092-8674(00)80434-1

[bib21] Liu X, Kim CN, Yang J, Jemmerson R, Wang X (1996) Induction of apoptotic program in cell-free extracts: requirement for dATP and cytochrome c. Cell 86: 147–157 8689682 10.1016/s0092-8674(00)80085-9

[bib22] Mack FA, Rathmell WK, Arsham AM, Gnarra J, Keith B, Simon MC (2003) Loss of pVHL is sufficient to cause HIF dysregulation in primary cells but does not promote tumor growth. Cancer Cell 3: 75–88 12559177 10.1016/s1535-6108(02)00240-4 PMC4120823

[bib23] Mandriota SJ, Turner KJ, Davies DR, Murray PG, Morgan NV, Sowter HM, Wykoff CC, Maher ER, Harris AL, Ratcliffe PJ, Maxwell PH (2002) HIF activation identifies early lesions in VHL kidneys: evidence for site-specific tumor suppressor function in the nephron. Cancer Cell 1: 459–468 12124175 10.1016/s1535-6108(02)00071-5

[bib24] Marsden VS, O'Connor L, O'Reilly LA, Silke J, Metcalf D, Ekert PG, Huang DC, Cecconi F, Kuida K, Tomaselli KJ, Roy S, Nicholson DW, Vaux DL, Bouillet P, Adams JM, Strasser A. (2002) Apoptosis initiated by Bcl-2-regulated caspase activation independently of the cytochrome *c*/Apaf-1/caspase-9 apoptosome. Nature 419: 634–637 12374983 10.1038/nature01101

[bib25] Martinou I, Desagher S, Eskes R, Antonsson B, Andre E, Fakan S, Martinou JC (1999) The release of cytochrome *c* from mitochondria during apoptosis of NGF-deprived sympathetic neurons is a reversible event. J Cell Biol 144: 883–889 10085288 10.1083/jcb.144.5.883 PMC2148194

[bib26] Maxwell PH, Wiesener MS, Chang GW, Clifford SC, Vaux EC, Cockman ME, Wykoff CC, Pugh CW, Maher ER, Ratcliffe PJ (1999) Nature 399: 271–275 10353251 10.1038/20459

[bib27] Motzer RJ, Bander NH, Nanus DM (1996) Renal-cell carcinoma. N Engl J Med 335: 865–875 8778606 10.1056/NEJM199609193351207

[bib28] Nagai T, Yamada K, Kim HC, Kim YS, Noda Y, Imura A, Nabeshima Y, Nabeshima T (2003) Cognition impairment in the genetic model of aging klotho gene mutant mice: a role of oxidative stress. FASEB J 17: 50–52 12475907 10.1096/fj.02-0448fje

[bib29] Newmeyer DD, Ferguson-Miller S (2003) Mitochondria: releasing power for life and unleashing the machineries of death. Cell 112: 481–490 12600312 10.1016/s0092-8674(03)00116-8

[bib30] Piret JP, Mottet D, Raes M, Michiels C (2003) Is HIF-1alpha a pro- or an anti-apoptotic protein? Biochem Pharmacol 64: 889–892 10.1016/s0006-2952(02)01155-3 12213583

[bib31] Presti JC, Rao PH, Chen Q, Reuter VE, Li FP, Fair WR, Jhanwar SC (1991) Histopathological, cytogenetic, and molecular characterization of renal cortical tumors. Cancer Res. 51: 1544–1552 1671759

[bib32] Silke J, Vaux DL (2001) Two kinds of BIR-containing protein – inhibitors of apoptosis, or required for mitosis. J Cell Sci 114: 1821–1827 11329368 10.1242/jcs.114.10.1821

[bib33] Soengas MS, Capodieci P, Polsky D, Mora J, Esteller M, Opitz-Araya X, McCombie R, Herman JG, Gerald WL, Lazebnik YA, Cordon-Cardo C, Lowe SW (2001) Inactivation of the apoptosis effector Apaf-1 in malignant melanoma. Nature 409: 207–211 11196646 10.1038/35051606

[bib34] Strasser A, Harris AW, Bath ML, Cory S (1990) Novel primitive lymphoid tumours induced in transgenic mice by cooperation between myc and bcl-2. Nature 348: 331–333 2250704 10.1038/348331a0

[bib35] Tamm I, Trepel M, Cardo-Vila M, Sun Y, Welsh K, Cabezas E, Swatterthwait A, Arap W, Reed JC, Pasqualini R (2003) Peptides targeting caspase inhibitors. J Biol Chem 278: 14401–14405 12538646 10.1074/jbc.M210133200

[bib36] Vaux DL, Cory S, Adams JM (1988) Bcl-2 gene promotes haemopoietic cell survival and cooperates with c-myc to immortalize pre-B cells. Nature 335: 440–442 3262202 10.1038/335440a0

[bib37] Vaux DL, Strasser A (1996) The molecular biology of apoptosis. Proc Natl Acad Sci USA 93: 2239–2244 8637856 10.1073/pnas.93.6.2239 PMC39779

[bib38] Verhagen AM, Coulson EJ, Vaux DL (2001) Inhibitor of apoptosis proteins and their relatives: IAPs and other BIRPs. Genome Biol 2, REVIEWS3009 1–3003.10 10.1186/gb-2001-2-7-reviews3009 PMC139420 11516343

[bib39] Wang S, Vrana JA, Bartimole TM, Freemerman AJ, Jarvis WD, Kramer LB, Krystal G, Dent P, Grant S (1997) Agents that down-regulate or inhibit protein kinase C circumvent resistance to 1-beta-D-arabinofuranosylcytosine-induced apoptosis in human leukemia cells that overexpress Bcl-2. Mol Pharmacol 52: 1000–1009 9396780 10.1124/mol.52.6.1000

[bib40] Wu XX, Mizutani Y, Kakehi Y, Yoshida O, Ogawa O (2000) Enhancement of Fas-mediated apoptosis in renal cell carcinoma cells by adriamycin. Cancer Res 60: 2912–2918 10850437

[bib41] Yao M, Shuin T, Misaki H, Kubota Y (1988) Enhanced expression of c-myc and epidermal growth factor receptor (C-erbB-1) genes in primary human renal cancer. Cancer Res 48: 6753–6757 2460228

[bib42] Zou H, Li Y, Liu X, Wang X (1999) An APAF-1Cytochrome *c* multimeric complex Is a functional apoptosome that activates procaspase-9. J Biol Chem 274: 11549–11556 10206961 10.1074/jbc.274.17.11549

